# Hardware Implementations of a Deep Learning Approach to Optimal Configuration of Reconfigurable Intelligence Surfaces

**DOI:** 10.3390/s24030899

**Published:** 2024-01-30

**Authors:** Alberto Martín-Martín, Rubén Padial-Allué, Encarnación Castillo, Luis Parrilla, Ignacio Parellada-Serrano, Alejandro Morán, Antonio García

**Affiliations:** 1 eesy-Innovation GmbH, 82008 Unterhaching, Germany; alberto.martin@eesy-innovation.com; 2 Department of Electronics and Computer Technology, University of Granada, 18071 Granada, Spain; rubenpadial@ugr.es (R.P.-A.); encas@ugr.es (E.C.); lparrilla@ditec.ugr.es (L.P.); 3 Department of Signal Theory, Telematics and Communications, University of Granada, 18071 Granada, Spain; parellada@ugr.es; 4 Department of Industrial Engineering & Construction, University of Balearic Islands, 07120 Palma, Spain; a.moran@uib.es

**Keywords:** 6G, reconfigurable intelligent surfaces, artificial intelligence, neural networks, FPGA

## Abstract

Reconfigurable intelligent surfaces (RIS) offer the potential to customize the radio propagation environment for wireless networks, and will be a key element for 6G communications. However, due to the unique constraints in these systems, the optimization problems associated to RIS configuration are challenging to solve. This paper illustrates a new approach to the RIS configuration problem, based on the use of artificial intelligence (AI) and deep learning (DL) algorithms. Concretely, a custom convolutional neural network (CNN) intended for edge computing is presented, and implementations on different representative edge devices are compared, including the use of commercial AI-oriented devices and a field-programmable gate array (FPGA) platform. This FPGA option provides the best performance, with ×20 performance increase over the closest FP32, GPU-accelerated option, and almost ×3 performance advantage when compared with the INT8-quantized, TPU-accelerated implementation. More noticeably, this is achieved even when high-level synthesis (HLS) tools are used and no custom accelerators are developed. At the same time, the inherent reconfigurability of FPGAs opens a new field for their use as enabler hardware in RIS applications.

## 1. Introduction

The future of mobile communications appears to be highly promising, as it is marked by the emergence of new use cases and complex demands. A forecast by the International Data Corporation (IDC) estimates that there will be 55.7 billion Internet of Things (IoT) devices in 2025, capable of generating 80 zettabytes (ZB) of data [1]. Meanwhile, after years of diligent research and development, the initial commercial rollout of the 5th generation (5G) mobile communication standard was successfully concluded in June 2018. At this moment, new 5G network deployments are underway in more than 30 countries [2], with a large offer of commercial 5G-compatible mobile devices. This significant advancement marks the beginning of a new era for mobile communications, presenting three distinct use cases with differing demands: enhanced mobile broadband, ultra-reliable and low-latency communications, and massive machine-type communications. Notably, the standardization process underscores a crucial realization: no single enabling technology exists that can fully accommodate all the diverse application requirements of 5G wireless networks [3].

Taking into consideration the discussion above, in order to meet future needs, experts in the field have already embarked on research into 6th generation (6G) wireless communications, which will provide possibilities for multiscale global connectivity and distributions [4]. There are several emerging trends around the idea of this new generation, including the development of new antenna technologies or the inclusion of artificial intelligence (AI) techniques into intelligent network systems [5], which will inevitably introduce communication engineering challenges. These challenges will require the exploration and adoption of radically new communication paradigms, particularly at the physical layer [3].

Traditionally, the propagation medium of wireless communications has been understood as a randomly behaving entity between the transmitter and the receiver. This randomness implies uncontrollable interactions of the transmitted radio waves that degrade the quality of the received signal. However, in recent years, a new technology with the potential of changing this by adding the possibility of controlling the wireless channel itself has appeared. This technology is the use of reconfigurable intelligent surfaces (RIS) [3,6,7,8,9], also referred to in the literature as intelligent reflecting surfaces (IRS) [10] or large intelligent surfaces (LIS) [11].

RIS are planar surfaces composed of an array of cells built of reflecting materials. RIS can be affixed to flat surfaces, such as walls or buildings, with the aim of redirecting radio frequency signals to the desired locations. Each RIS cell can be individually configured, generating a specific arrangement that will reflect the incident signal towards the target device. In order to enable more efficient communication, the signal must be transmitted to the receptor system without encountering obstacles or necessitating signal repeaters, which, in turn, results in an increase in power consumption. Therefore, it is this ability, just by reconfiguring the reflecting surface, to redirect the incident signal to the target devices that positions RIS as a key technology for the green transition to 6G communications [5].

The optimization problem associated with the computation of the most suitable RIS configuration for a certain scenario may require significant real-time data processing, which is not always possible. In [9], the non-dominated sorting genetic algorithm (NSGA-II) is used to derive the RIS configuration by maximizing the amount of energy concentrated in the particular region of space of the RIS that enables the desired redirection of the beam. This method begins with an initial RIS configuration and iteratively changes random parts until the resulting RIS reaches the target signal amplitude, which makes the process time-consuming and unsuitable for edge computing applications (the computation of a single configuration with this algorithm may take up to 3 min on a 20-core Intel^®^ Xeon^®^ server). Thus, artificial intelligence, which has achieved significant breakthroughs in big-data processing systems, has been considered in the literature as an option to address this optimization [12]. Furthermore, edge computing is emerging as a solution for decentralized data processing, both reducing the latency associated with cloud computing and aiding the achievement of real-time processing [13]. Considering these facts, this work introduces a novel approach to compute the configuration of RIS devices, illustrated with a 1-bit 15 × 15 RIS example. Thus, this proposal makes use of a convolutional neural network (CNN) specifically designed and trained to compute the phase shift for each RIS cell according to certain input features based on a representation of the desired redirection angle for the incident signal. Furthermore, this CNN is optimized for deployment on different devices including field-programmable gate arrays (FPGAs), which are an ideal solution to implement AI accelerators as well as to optimize performance at the edge computing level [14]. Hence, this work showcases the implementation of an ad hoc CNN on various devices to address the RIS optimization challenge. Its main contributions reside in the ability of this CNN to be adapted to different data flows and target devices, and the illustration of the advantages of the combination of AI techniques and FPGA devices as the target technology to enable the adoption of RIS for 6G deployment.

The rest of the manuscript is organized as follows. Section 2 provides a brief overview of RIS devices, their applications, and the diverse architectures currently under study. It also discusses the basics of using AI techniques to solve the optimization problem for obtaining a RIS configuration according to the signal redirection objective. Furthermore, it explores the relevance of computing this on the edge, as well as the current devices in this field. In Section 3, the databases on use and the neural network (NN) design process are described. Section 4 details the NN implementation method on different devices. Finally, in Section 5, the results of the deployment of the resulting CNN are presented and compared using different devices, and the conclusions are summarized in Section 6.

## 2. Edge Computing for RIS

### 2.1. Reconfigurable Intelligent Surfaces

**Figure 1:**
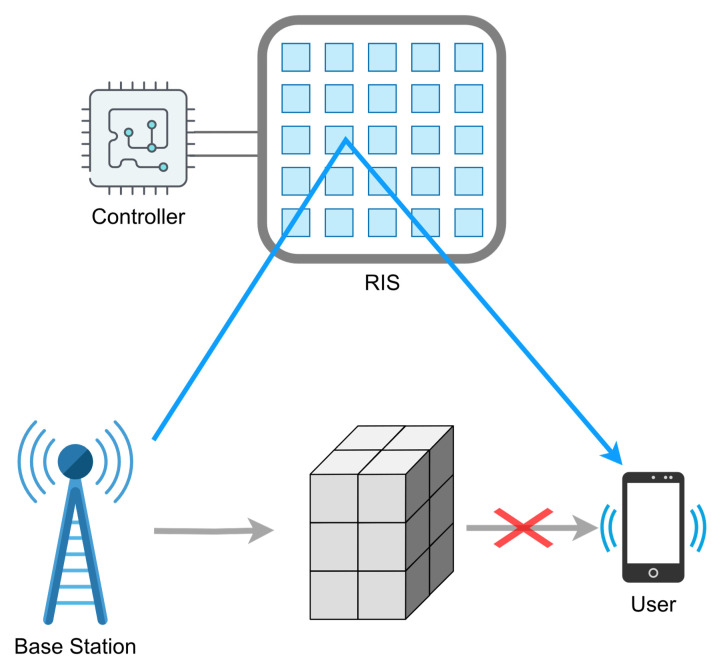
Usage of RIS to create a virtual LOS.

RIS technology emerges as a key player in shaping the future of wireless communications. At 6G frequencies, it is highly probable that signals are absorbed, reflected, or scattered by common urban and rural elements such as buildings, hills, and vehicles. Thus, the environment can become hostile to signal transmission. In such a scenario, maintaining a direct line-of-sight (LOS) between the emitter or base station (BS) and the users is crucial, and this is precisely how RIS technology becomes a key feature in the 6G era [8]. The way this is accomplished is by effectively establishing a virtual LOS [15]. RIS can be strategically placed in the radio channel between the transmitter and the receiver, as shown in Figure 1, so the RIS cell configuration is adjusted to purposefully reflect the signal toward the user’s receptor.

RIS technology does not only bring the advantage of preventing signals from being blocked by obstacles, but it also has the potential to establish a secure network by simultaneously increasing the received signal power for the intended user and minimizing any information leakage to potential eavesdroppers [15]; at the same time, tracking the position of the the target user ensures uninterrupted communications despite users moving around [16]. Furthermore, there are additional beamforming applications in which the incident signal is divided into multiple beams and redirected towards multiple users. For instance, the potential benefits that RIS could bring to multicast networks or IoT networks have been explored in the literature [15]. RIS applications are thus almost limitless.

An RIS can be defined as an array functioning as an antenna, typically built using either metamaterials or conventional patch-array antennas equipped with rapid electronic-switching capabilities. These arrays have the capacity to control electromagnetic waves by enabling anomalous reflection, refraction, polarization transformation, and various other functionalities. In this context, our focus is on RIS configured as anomalous reflective and/or refractive surfaces capable of tailoring the propagation environment by directing signals to desired directions through reflection and/or refraction [5]. Depending on the RIS application and the throughput required, various hardware configurations and operational modes come into play. In terms of cell architecture, RIS can be continuous, in which the finite surface is made up of a virtually infinite number of elements, or discrete, where a limited number of independent elements are configured to achieve the desired phase shift [17].

The number of elements is closely related to the resolution achieved in the target angle by the RIS device and depends on the number of phase shifts each cell can perform. The simplest cell is a binary cell, which allows two-phase shifts, 0^∘^ and 180^∘^, coded in a single bit. In any case, the availability of more phase-shift levels implies better resolution at the cost of higher complexity in the computational problem of RIS configuration.

### 2.2. Why Deep Learning and Not Other Approaches

Another critical aspect is the optimization method to compute the precise phase shift that each cell must provide in order to meet the redirection demands for a particular RIS or a particular application. Numerous mathematical approaches have been detailed in the literature to optimize phase shifts, many of which rely on iterative calculations. However, these methods are both resource-intensive and time-consuming. Deep learning (DL) techniques involve feeding an algorithm with a vast amount of data, enabling it to solve a complex problem automatically based on the experience gained during the learning process [18]. As a result, the required computation resources can be reduced.

In recent years, the growing utilization of AI has demonstrated its potential to address this optimization challenge with reduced time and resource usage [12]. The strategy used in both the arithmetical and AI methods involves calculating a continuous phase shift to be implemented in each cell. If discrete phase shifts must be used due to design or hardware constraints, the preferred approach is to compute the continuous phase shift and then quantize it based on the available discrete levels in the design [12].

In this work, the input data format, discussed further in Section 3, is a color image with three separate 2D arrays, with each array corresponding to one of the RGB (red, green, blue) color channels. This makes CNNs perfectly suitable for processing these data [18]. The use of CNNs in applications such as face recognition is widely known, but they are extensively used as an advanced alternative to classical image processing in applications such as food or character recognition [19], where images are processed to extract certain features. Considering all of the above, CNNs can be considered as a suitable option to process target angle-derived information and thus optimally obtain the desired RIS configurations.

### 2.3. Why on the Edge

With the rapid evolution of technology, the increasing number of data-transmitting devices, including IoT devices, and the resulting substantial increase in the volume of data sent to the cloud for processing, edge computing has emerged as a pivotal paradigm nowadays. Instead of sending a large amount of data to a central server, data are processed locally, just where sensor or actuator devices are deployed. Consequently, edge devices, placed close to data sources and end-users, play a crucial role in processing and analyzing data locally, thus mitigating the challenges posed by latency, bandwidth, and privacy concerns [13].

This shift towards edge computing is also a consequence of the current state of data science, which demands the processing of vast quantities of data during both the learning and inference processes for artificial neural networks (ANNs). In this context, edge computing holds the potential to enhance performance significantly, enabling efficient AI computational acceleration through edge devices suitable for AI processing such as central processing units (CPUs), graphical processing units (GPUs), tensor processing units (TPUs), FPGAs, or dedicated application-specific integrated circuits (ASICs) [20]. A clear example of this is the emergence of embedded GPU-based technologies, also referred to as neural processing units (NPUs), that several smartphone manufacturers are integrating into their devices to process data with AI algorithms on the edge [21].

This study proposes a novel approach to compute RIS configurations from data derived from target angles, in which a signal must be redirected using a RIS device whose configuration is inferred by AI algorithms. This derived information can contain large volumes of data and, furthermore, the computational load can be intensified as the size of the target RIS increases. Consequently, sending all these data to be processed in a server and having the RIS configuration sent back to the device or devices modifying the RIS setup could result in significant data bandwidth, along with notable data latency. As a result, this approach might not be efficient in meeting real-time requirements. Considering all this, the use of edge devices becomes essential to mitigate latency and reduce data bandwidth effectively.

### 2.4. Target Edge Devices

Numerous devices have been explored in the literature to enhance the performance of edge computing. These devices are designed with the aim of optimizing various aspects of edge computing, such as latency reduction, enhanced processing capabilities, or improved energy efficiency. GPUs are among the devices that are more generally used to compute AI on the edge. GPUs were originally developed and architected to process images and videos. Comprising multiple parallel processors, GPUs facilitate parallelization, i.e., breaking down complex problems into smaller tasks that can be simultaneously computed. This feature makes GPUs suitable for AI training and inference, where a vast amount of data and calculations are needed, and the parallel computing capacity significantly speeds up the process [14].

In recent years, GPUs have played a pivotal role in accelerating AI tasks. However, GPUs imply more power consumption than other specific devices aimed for AI, such as TPUs, or devices with a hardware configuration specifically designed for the goal, such as FPGAs or ASIC devices. For this reason, along with the booming interest in AI, Google developed a device specifically intended to run DL models with an exceptional degree of efficiency. These devices are known as TPUs, which comprise arrays of multiplication units. Initially designed for cloud computing, the first versions from Google, TPU1 and TPU2, were enormous servers to compute data in a data center. However, the evolving trend towards edge computing has driven the evolution to edge TPUs, designed to meet power consumption and size requirements while delivering high-performance acceleration [14]. One such example of these devices is Google Coral, which has been chosen to implement the neural network developed in this study, thereby enabling a comparison with other target devices.

The final devices considered in this study are FPGAs. FPGAs are reconfigurable devices that provide the capability to implement customized hardware designs. Due to their inherent flexibility, they can be applied to a wide range of fields, and, notably, recent studies have positioned them as key components in the realm of AI science [14]. The development of tailored hardware to compute the target NN and the required operations within an FPGA brings the benefit of optimizing and parallelizing the computation according to the design limit and the capacity of the target hardware device. Flexible architectures of FPGA devices not only offer the advantage of optimizing NN architectures, but also enable the implementation of the additional features required in the final implementation. For instance, the development on FPGAs of digital control systems for reconfigurable antennas has been explored in the literature [22]. This approach opens up the possibility of implementing the RIS-cell control system along with the AI optimization algorithm to configure each RIS cell according to the desired redirection.

## 3. Methodology

As it was detailed above, the aim of this work is to develop a deep learning model able to estimate the optimal configuration of an RIS, which is illustrated with a 1-bit 15 × 15 RIS example. The approach that has been followed to achieve this goal is to consider this optimization as a classification problem, i.e., given an input derived from the target angle for the beam redirection of the RIS, each RIS cell is classified into two classes, 0 or 1 ($0^{\circ }$ or $180^{\circ }$ phase-shift), depending on the configuration needed to create the desired redirection. Thus, each cell is activated or deactivated to change the signal phase according to its classification result. This strategy is only valid when the RIS resolution is coded in 1 bit; however, for larger resolutions, this approach would still be valid when adapted to the necessary number of bits per cell.

In order to obtain a model that estimates the optimal configuration of the RIS, it was decided to use a supervised learning approach to train the neural network. Therefore, it is necessary to have a correctly labeled dataset that serves as the ground truth, based on which the neural network is optimized and tested. In this work, radiation diagrams have been used as the input to the network. These diagrams show in which direction the signal reflected by the RIS has the greatest energy. For each of these diagrams, there is an associated RIS configuration, thus obtaining the pairs $\left(x,y\right)$ used for training. Section 3.1 will show a detailed explanation on how the dataset is generated.

To train the network, it is also required to take into consideration the fact that equivalent configurations for the RIS are possible. This means that, for a given target redirection of the incident wave in the RIS, two or more valid configurations are possible. The number of these valid solutions varies according to the number of bits used to represent the phase-shift. In order to address this, a custom loss function, which takes this fact into consideration for the training of the model, has been developed. Further details on the model architecture, custom loss function, training and validation, and evaluation are available in Section 3.2, Section 3.3, and Section 3.4, respectively.

### 3.1. Dataset Generation

The first step in developing an ANN involves the generation of a comprehensive dataset. In light of the points mentioned above, this work introduces a novel approach to compute the configuration of an RIS device. In this way, the input data must represent the direction in which the RIS is intended to redirect the incident wave. To do this, the resolution of the RIS has to be taken into account, given that, as mentioned above, the state of each of the cells is represented with just 1 bit. This, combined with the assumption that what impinges the RIS is a plane wave, causes the reflected wave not to have a main lobe, but rather two specular ones [23]. Furthermore, due to the periodicity of the RIS cells being 0.75λ, grating lobes will start to emerge in undesired directions when the direction of the reflected wave deviates significantly from that of the incident wave. Due to these side effects, it is not necessary to sweep all the possible direction values of the reflected wave when generating the dataset.

For the generation of the training dataset, the illumination of the RIS with the plane wave was set to be normal. In the spherical coordinate system, assuming that the RIS is positioned in the XY-plane, a sweep is performed in azimuth (ϕ) from $-89^{\circ }$ to $90^{\circ }$ and in elevation (θ) from $-25^{\circ }$ to $25^{\circ }$, with $1^{\circ }$ resolution in both cases. In this way, the space for z>0 where no grating lobes are expected is covered, and, as for z<0, no radiation is foreseen due to the nature of the RIS. For each of these directions, a pair $\left(x,y\right)$ of training values was generated, where the input of the network, *x*, is the desired direction of the reflected wave, and the output, *y*, is the optimal RIS configuration achieving that redirection. The total number of training pairs is thus 9180.

**Figure 2:**
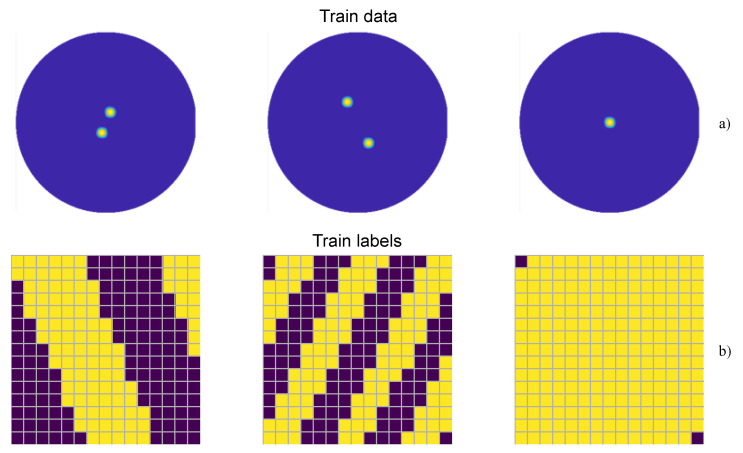
Samples extracted from the training dataset: (**a**) train images, (**b**) train labels.

In order to represent the desired direction for the reflected wave, the information is presented in the form of a radiation diagram, which is an image of 343 × 342 pixels. Figure 2a illustrates three samples of the radiation diagrams. These images show the space distribution of the energy of the signal, therefore indicating in which direction the signal has the greatest energy. For this, a UV mapping was performed, so that a transformation of the ϕ/θ angles to their corresponding u/v space coordinates was made. This coordinate transformation is detailed in Equation (1):

(1) $$\begin{array}{c} \begin{array}{cc} u & =\sin\theta \cos\phi \\ v & =\sin\theta \sin\phi . \end{array} \end{array}$$

The label for each of these images is represented as an array of 225 elements (since the size of the RIS is 15 × 15), with each element being the phase-shift of each of the cells of the RIS. Three examples of the resulting RIS configuration are represented in Figure 2b. These labels were obtained through a brute-force ad hoc algorithm characterized as follows:

- It takes as a reference the amount of energy in the desired direction with a random configuration of 0/1. Now, cell by cell, their states are inverted, and then the energy is again checked. If it has increased, the inversion is maintained; if not, it is reverted. When all the elements have been processed, new sweeps are performed until the stopping criterion is met: at the end of a sweep, less than 10% of the element’s states have been inverted. This threshold is set because the computational cost of conducting a new sweep does not justify the marginal improvement in the energy value.
- Although the algorithm converges relatively quickly, the computational cost is high and sustained over time and not suitable for real-time calculations.

With all this in mind, the use of an ANN-based algorithm to calculate the optimal configuration of the RIS is justified, since these algorithms can be accelerated on different platforms, making it feasible to perform these calculations in real time.

### 3.2. Model Architecture Design

CNNs are nowadays one of the most effective tools for computer vision applications, such as object detection [24], image recognition [25], or image classification [26]. The way they process data makes manual feature engineering unnecessary, as is usually the case with traditional algorithms, since CNNs achieve this feature learning from large datasets. As it was detailed in Section 3.1, the input data in this work are images representing the space distribution of the energy of the wave, which makes CNNs especially suitable for this application.

**Figure 3:**
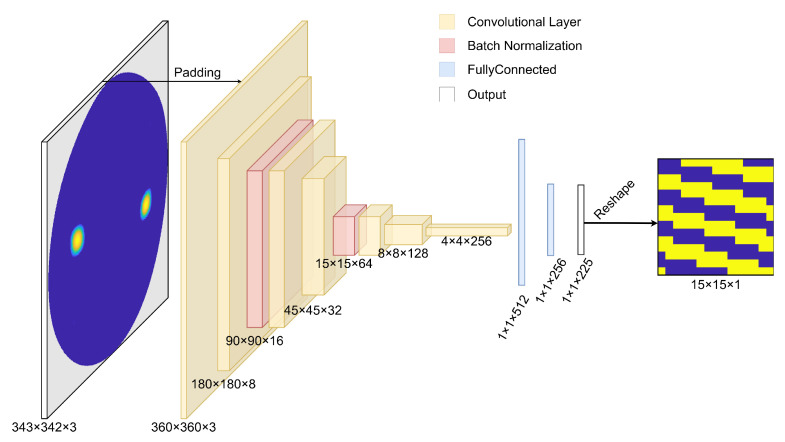
Convolutional neural network architecture diagram.

The NN model architecture design has been based on some well-known CNNs in the literature, which have also been implemented on FPGAs [27]. One of them is the LeNet-5 model, a modification of the classic LeNet [28], which has been used in many application scenarios, like medical diagnosis [29], signal processing [30], or image segmentation [31]. It is a simple and efficient CNN, which offers great performance in some of the cases. However, this model does not deliver strong performance in this particular application. As a result, a modified version of the OverFeat model [32], winner of the ImageNet Large Scale Visual Recognition Challenge 2013, was considered, and it was adjusted to the requirements of this specific RIS problem. The number of layers and the architecture’s complexity were determined through experimentation and validation on a separate validation set, and the final architecture design is shown in Figure 3.

The first layer of the network is a Lambda layer, whose purpose is to normalize the image so that each pixel has values in the range [0, 1]. It must be noted that the original 343 × 342 input image is first padded to 360 × 360 to match this Lambda layer better. After that, a set of convolutional layers is applied, whose aim is to reduce the dimension (width and height) of the original image progressively and increase the feature dimension. The activation functions are ReLU layers, which are commonly used in CNNs. TensorFlow [33] and Keras [34], which are software tools widely used in data science, were chosen for the development of the CNN.

During training of a deep neural network, one of the possible difficulties that can arise is that the distribution of the inputs to layers deep in the network may change after each mini-batch when the weights are updated. This can cause the learning algorithm to chase a moving target. To solve this problem, two batch normalization layers are placed between the inner convolutional layers. These layers apply a normalization by mean and standard deviation, plus re-scaling (γ) and biasing (β) parameters, as shown in Equation (2). These two parameters are learnable parameters and will enable the accurate normalization of each batch during training:

(2) $$y=\frac{x-E[x]}{Var[x]+\epsilon }\gamma +\beta .$$

The final stage of the network is composed of two fully connected layers, which apply a multiplication by a weight matrix and the sum of a bias to obtain the classification result from the features extracted by the convolutional layers. The activation function of the last fully connected layer is a Sigmoid, since its output range is limited to the interval [0, 1] and can be related to a probabilistic value. If the outcome of the Sigmoid function is greater than 0.5, then the classification result will be in the 1-class, or positive, while the remaining outcomes will be classified as 0 (negative class). Table 1 shows a complete summary of the model architecture.

### 3.3. Custom Loss Function

**Figure 4:**
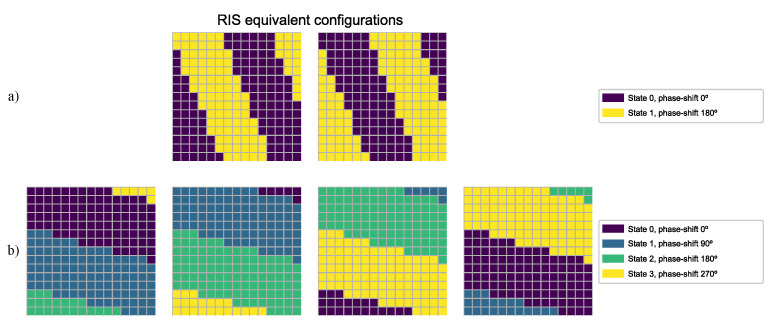
RIS equivalent configurations for different resolutions: (**a**) states represented with 1 bit, (**b**) states represented with 2 bits.

One of the considerations in the description above, as also mentioned in the introduction to this section, is the fact that there are equivalent configurations that cause the RIS to redirect the incident waves in the same direction. Concretely, for any given RIS configuration, if the state of every cell is modified by the same phase-shift offset, the resulting configuration will be equivalent to the initial one. Thus, if the phase-shift is discrete and represented with *k* bits, there will be $N=2^{k}$ equivalent configurations for each possible combination of cells, since only $2^{k}$ phase-shift states are possible for each cell. Figure 4 shows two examples of equivalent configurations. It can be seen that, with 1-bit resolution, the equivalent configuration of any combination of cells is thus just its logical NOT, as the only applicable phase-shift offset is $180^{\circ }$, i.e., each cell is switched to the other possible state. In the case of 2-bit resolution, 4 equivalent configurations are possible, which correspond to $90^{\circ }$ offsets.

The loss function, or cost function, computes the “distance” between the current output of the model and the expected output, so the target is to minimize it during training. The classical loss function that is applied in binary classification problems is called *binary cross-entropy*, and it is shown in Equation (3):

(3) $$L=-\frac{1}{N}\sum_{i=1}^{N}y_{i}\cdot \log\left({\hat{y}}_{i}\right)+\left(1-y_{i}\right)\cdot \log\left(1-{\hat{y}}_{i}\right),$$

where *N* is the number of samples in the batch, $y_{i}$ is the expected output, and $\hat{y_{i}}$ is the estimated one. In order to use this function, it will be necessary to adapt it, since in this case there are multiple binary outputs, one for each cell. However, the main problem with this function is that the distance between two equivalent configurations is the maximum when it should be the minimum. In order to overcome this, a custom loss function was developed, which takes into consideration the expected output and its logical NOT, taking the minimum cross-entropy between them. This loss function is shown in Equations (4)–(6):

(4) $$L_{1_{i}}=-\frac{1}{M}\sum_{j=1}^{M}y_{ij}\cdot \log\left({\hat{y}}_{ij}\right)+\left(1-y_{ij}\right)\cdot \log\left(1-{\hat{y}}_{ij}\right)$$

(5) $$\begin{array}{cc} L_{2_{i}}= & -\frac{1}{M}\sum_{j=1}^{M}{\bar{y}}_{ij}\cdot \log\left({\hat{y}}_{ij}\right)+\left(1-{\bar{y}}_{ij}\right)\cdot \log\left(1-{\hat{y}}_{ij}\right)\\ \; & =-\frac{1}{M}\sum_{j=1}^{M}\left(1-y_{ij}\right)\cdot \log\left({\hat{y}}_{ij}\right)+y_{ij}\cdot \log\left(1-{\hat{y}}_{ij}\right) \end{array}$$

(6) $$L=\frac{1}{N}\sum_{i=1}^{N}\min\left(L_{1_{i}},L_{2_{i}}\right),$$

where *M* is the number of cells of the RIS and *N* is again the number of samples in the batch, while $\bar{y_{ij}}$ is the logical NOT of the expected output.

### 3.4. Model Training and Evaluation

The Adam optimizer [35] was used for the training of the model. It is an extended version of the classic stochastic gradient descent that has demonstrated good results, with a learning rate of 0.001. For the batch, there is a trade-off between large and small sizes. When it is large, there is a degradation in the quality of the model. On the other hand, when it is too small, the noise of the gradient is increased. In this study, it was observed that 32 samples per batch offers the best results, achieving good results with 100 epochs.

For the same reason that justifies the need for a custom loss function, to evaluate our model it was also necessary to use a custom accuracy computation that takes into consideration equivalent configurations. This is shown in Equation (7):

(7) $$accuracy\left(y,\hat{y}\right)=\max\left(\frac{1}{M}\sum_{j=1}^{M}I\left({\hat{y}}_{j}=y_{j}\right),\frac{1}{M}\sum_{j=1}^{M}I\left({\hat{y}}_{j}={\bar{y}}_{j}\right)\right).$$

Here, the same nomenclature is followed, with ${\hat{y}}_{j}$ being the *j*-th cell of the estimated configuration, and $y_{j}$ and ${\bar{y}}_{j}$ the *j*-th cell of the expected configuration and its logical NOT (the equivalent configuration), respectively. With this combination of train hyperparameters, the obtained accuracy is 98.88%.

## 4. Implementation

Edge computing is becoming a viable alternative as technology advances and more computation resources are available within edge devices and their inherent limitations. While a wide variety of options are available, the most prevalent devices used in edge computing were discussed in Section 2.4. Thus, and in order to facilitate a comparison among these edge devices, one representative example from each category was selected in this study. The ROCK 4C Plus [36] is a single board computer (SBC) used in this study to infer the NN on a CPU. In the case of GPUs, the study focuses on the NVIDIA Jetson Nano platform [37], an SBC combining a CPU and a GPU to enhance machine learning computations. The Google Coral [38], on the other hand, features a TPU, while the Intel^®^ Arria^®^ 10 SX SoC Development Kit [39] board is used as the representative FPGA device in this comparative analysis. Their respective specifications are summarized in Table 2.

### 4.1. Neural Network Adaptation

To ensure a reliable comparison, the initial NN design was adjusted to find a compromise for compatibility with the different platforms under study. This approach involves deploying and executing the exact same neural network architecture on each of the aforementioned devices. Respective frameworks are used to tailor and perform AI applications on each device, resulting in different approaches for implementing the same neural network on each platform. However, it is important to note that all of these implementations sprout from the pretrained NN model, and inference and testing were performed with exactly the same dataset.

**Figure 5:**
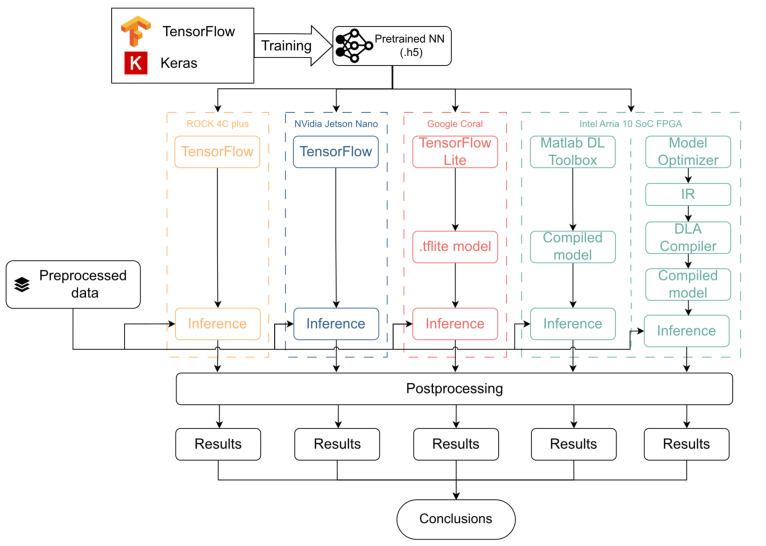
Implementation workflow diagrams for different platforms.

The primary limitation in the initial NN design stems from its implementation on FPGA, which is constrained by the frameworks in use. Notably, the Reshape and Lambda layers are not supported by the tools employed for NN implementation. Consequently, these layers were excluded from the initial design for all the selected devices, and a new training process was conducted using the revised architecture. The resulting neural network remains identical in terms of architecture, but these excluded functions must now be executed externally to the NN deployment. They can thus be regarded as preprocessing and postprocessing functions. The Lambda layer is responsible for normalizing the input image, while the Reshape operation aligns the output data dimensions to the requirements of the RIS. The result of this new training process is a new pretrained NN, which is now compatible with all the devices under study. It achieves an accuracy of 99.88% across the dataset when implemented and tested in the TensorFlow framework, as expected from the results in Section 3.4. The general workflow for implementation and testing over the different platforms is depicted in Figure 5, while it must be noted that some of the test platforms are compatible with the INT8 data type. Thus, the model has also been quantized to INT8, and the corresponding training was carried out in order to optimize this quantized model. Both the FP32 and INT8 models are implemented in the different platforms according to their suitability to these two data types, and the results over these test platforms are presented and discussed in Section 5.

### 4.2. CPU—ROCK 4C Plus

Despite including a GPU alongside the CPU, the ROCK 4C Plus board was chosen to run the target neural network on the lower-capability CPU. This selection was made as a reference point for the comparison, and to enable benchmarking with the other devices in this study, which are more specialized for AI applications. This approach ensures a well-rounded performance assessment across a range of devices with varying capabilities and intended use cases.

Since the neural network is executed using the FP32 data type and the architecture is compatible with the TensorFlow framework, no modifications are required for inference on this device. Therefore, the pretrained neural network is executed directly with TensorFlow to predict the RIS configuration for the previously preprocessed dataset and to compute accuracy, as described in Section 3.4. Additionally, the platform can also support INT8, so this quantized model is also tested on the ROCK 4C Plus, as will be discussed later.

### 4.3. GPU—NVIDIA Jetson Nano

The NVIDIA Jetson Nano is a compact yet powerful solution specifically designed for AI applications. This SBC combines a CPU with the NVIDA Maxwell architecture GPU with 128 NVIDIA CUDA^®^ cores [40], thus allowing highly parallel computations and making it a suitable choice for AI development. The NVIDIA Jetson Nano platform is also compatible with the TensorFlow framework for inference, so a similar approach to that in Section 4.2 was adopted. The key distinction lies in the fact that this SBC includes a GPU equipped with NVIDIA CUDA^®^ cores. As a result, the NN takes advantage of this GPU acceleration for execution, although only the FP32 model is supported.

### 4.4. TPU—Google Coral

Implementing neural networks on the Google Coral platform offers a powerful and efficient solution for AI inference tasks. Google Coral is known for its Edge TPU [41], which is a dedicated hardware accelerator designed to accelerate machine learning workloads on edge devices. TensorFlow Lite is used to convert and optimize the pretrained NN for deployment on the Coral device. The AI model is then executed with TPU hardware-acceleration, and dataset outputs are predicted. However, the use of the TPU does require the NN to be quantized to the INT8 data type; otherwise, the NN would be executed exclusively by the ARM CPU cores. Thus, in the case of the Google Coral TPU, the INT8-quantized NN model is the only one tested in this platform.

### 4.5. FPGA—Intel^®^ Arria^®^ 10 SX SoC Development Kit

The NN implementation on FPGA devices is carried out using two different high-level synthesis (HLS) frameworks: the MATLAB ^®^ Deep Learning Toolbox^TM^ [42] version 14.6 and the MATLAB^®^ Deep Learning HDL Toolbox^TM^ [43] version 1.5, on the one hand, and the OpenVINO toolkit [44] along with the Intel^®^ FPGA AI Suite [45], on the other hand. The MATLAB^®^ R2023a revision is used in this study, while the Intel^®^ toolkit comprises the OpenVINO Toolkit version 2022.3 LTS and the Intel^®^ FPGA AI Suite version 2023.2, along with Quartus^®^ Prime Pro 22.4.0 [46]. The workflow for both sets of tools is detailed in the following.

#### 4.5.1. MATLAB^®^ Deep Learning HDL Toolbox^TM^

**Figure 6:**
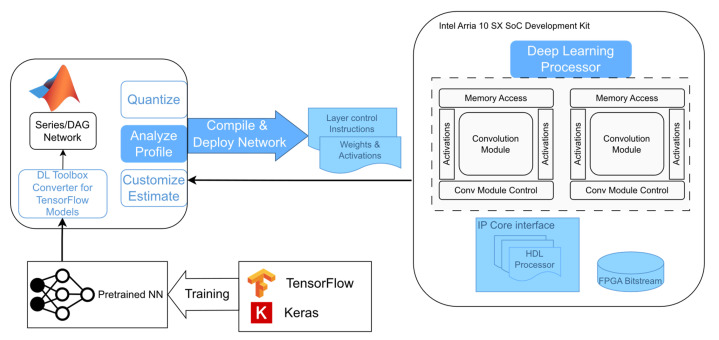
NN implementation workflow for the Intel^®^ Arria^®^ 10 SX SoC Development Kit device using the MATLAB^®^ Deep Learning HDL Toolbox^TM^.

The Deep Learning HDL Toolbox^TM^ provides a prebuilt image with the embedded software and the FPGA configuration file implementing the AI acceleration hardware and I/O peripherals. The workflow is illustrated in Figure 6 [47].

The implementation begins with the externally pretrained (using TensorFlow 2.6.0 and Keras 2.6.0) NN. This pretrained model is imported into MATLAB^®^ using the Deep Learning Converter for TensorFlow Models. However, as detailed above, the Lambda and Reshape layers are not supported by this Deep Learning HDL Toolbox^TM^. Additionally, the Sigmoid layer is supported by the tool but it is not specifically implemented in the prebuilt bitstream for the device in the Intel^®^ Arria^®^ 10 SX SoC Development Kit. Thus, as detailed in Section 4.1, the Lambda and Reshape layers are excluded from the NN for all platforms and are run as preprocessing and postprocessing operations in the host computers, while the Sigmoid activation layer is implemented in this case as software run in the Arria 10 SoC CPU.

**Figure 7:**
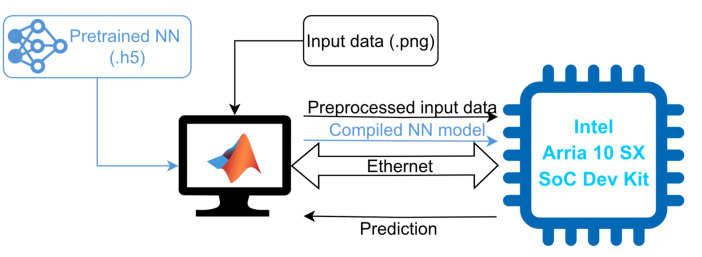
Intel^®^ Arria^®^ 10 SX SoC Development Kit test setup. The dataflow related to the NN accelerator creation and deployment is represented in blue, while the test dataflow is illustrated in black.

Once the neural network is imported and converted into a Series or DAG Network object in MATLAB^®^, the NN is compiled and deployed to the FPGA device using the MATLAB^®^ Deep Learning HDL Toolbox^TM^. It generates layer control instructions for executing the specific neural network with the provided accelerator, and the weights and activations are loaded into the device based on quantization level considerations. The NN can be compiled to make use of either the FP32 (single) data type, which is the resulting one after training and the same as that used for TensorFlow inference, or the INT8 quantization, which yields better performance at the cost of reduced accuracy. The compiled model and the preprocessed input data are transmitted from the host computer to the Intel^®^ Arria^®^ 10 SX SoC Development Kit device via the Ethernet link. The output is also received by the host computer using the same communication channel. The test setup is depicted in Figure 7. The Deep Learning HDL Toolbox^TM^ not only provides the model compilation and inference, but also provides data and tools to analyze and enhance NN performance on the target hardware and reports latency and throughput (frames-per-second, FPS). Table 3 provides an overview of the utilization of key FPGA resources for the deep learning accelerator implementations for both FP32 and INT8 data types, while the clock frequency of the DL processors is 200 MHz and 150 MHz, respectively, for these data types. A larger resource usage is noticeable in Table 3 for the INT8 implementation, which is explained by the use of more parallel convolution threads than what is possible with FP32.

#### 4.5.2. Intel^®^ FPGA AI Suite

**Figure 8:**
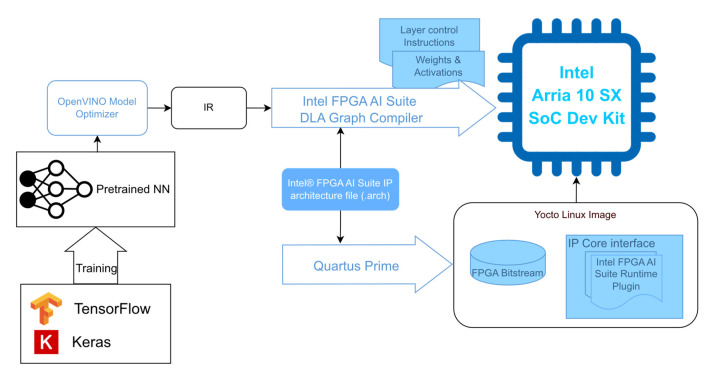
Intel^®^ FPGA AI Suite implementation workflow.

The Intel^®^ FPGA AI Suite tool flow works with the OpenVINO toolkit, which is an open-source project to optimize inference on a variety of hardware architectures. The pretrained TensorFlow/Keras model is fed to OpenVINO and converted into an intermediate representation (IR) format. This format encapsulates the neural network topology along with the weight and bias information. Subsequently, this IR model is optimized with the Intel^®^ FPGA AI Suite DLA Graph Compiler for inference on the target hardware, the Intel^®^ Arria^®^ 10 SX SoC Development Kit in this case. The workflow is illustrated in Figure 8. Simultaneously, the so-called CoreDLA IP core (deep learning accelerator IP core) is built for this target device accordingly to the configuration specified in the IP architecture file. The resulting bitstream is then integrated within the Yocto Linux system, alongside the FPGA AI Suite Runtime Plugin for inference.

The CoreDLA IP can be built using different sets of performance/area parameters, which include the possibility of using the FP16 data type. However, as it is not available for any of the other CPU-based platforms or the MATLAB^®^ Deep Learning HDL Toolbox^TM^, only implementations from the FP32 model are considered in this case, with the A10_Performance and A10_Generic architectures [48]. Thus, Table 4 shows a summary of the FPGA resource usage for these two Intel^®^ FPGA AI Suite implementations. It must be noted that these resources correspond to the CoreDLA IP as well as to all the peripherals, including a Nios^®^ V [49] instance, required for the communication between the CoreDLA IP and the HPS (hard processor system) CPU of the Arria 10 SoC device. Moreover, the CoreDLA IP is clocked in the two architectures at 100 MHz, even when frequencies above 200 MHz are supported in both cases. The comparison of Table 3 and Table 4 makes it clear that a more compact implementation is possible with the Intel^®^ FPGA AI Suite, although the MATLAB^®^ Deep Learning HDL Toolbox^TM^ allows users with practically no hardware background to implement and use the NN accelerators, as well as interact with them directly from the MATLAB^®^ user interface. Performance results for all the test platforms are discussed in Section 5.

## 5. Results

The different implementations have been described in Section 4, and accuracy and performance results are presented below for the different test platforms and devices.

### 5.1. Accuracy

**Figure 9:**
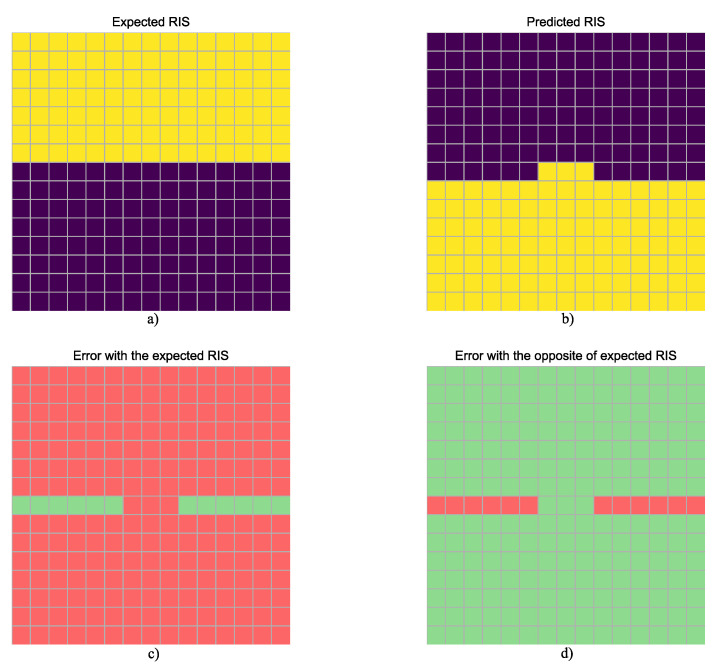
Inference example, Intel^®^ Arria^®^ 10 SX SoC Development Kit with Matlab^®^ Deep Learning HDL Toolbox^TM^ FP32 accelerator: (**a**) expected RIS, (**b**) inferred RIS, (**c**) error when matching the expected RIS and the inferred RIS, and (**d**) error when matching the opposite of the expected RIS and the inferred RIS (errors are shown in red in both (**c**,**d**), coincidences in green).

In order to illustrate how accuracy is computed, it must be noted that, once the platform in use was implemented, outputs were inferred for each input in the dataset. Thus, accuracy is calculated on the basis of the labels or expected RIS, considering that both the label and the opposite/complement are valid solutions, as discussed in Section 3.3. An example of this is illustrated in Figure 9, where the output is computed using the Intel^®^ Arria^®^ 10 SX SoC Development Kit, implementing the MATLAB^®^ Deep Learning HDL Toolbox^TM^ FP32 accelerator, for the test element with index 94; the predicted RIS is compared with both the expected RIS and its opposite. In this case, the opposite RIS is predicted with 12 incorrect cells, resulting in an accuracy of 94.67% for this particular element in the dataset and this hardware accelerator. The hit rate is then averaged across all predictions for each accelerator under study. Table 5 provides a summary of the accuracy results for the different platforms and frameworks, as well as for both FP32 and INT8 data types. The results in Table 5 confirm that all the tested platforms/implementations are performing adequately and in the same range of accuracy, always keeping in mind the slight reduction in accuracy that can be expected from the INT8-quantized implementations. Regarding the FP32 implementations, all of them provide the same accuracy, which also matches the value derived from the model evaluation in Section 3.4. This confirms the validity of all implementations, as there should be no noticeable deviation from the software evaluation when floating-point is used. The INT8-quantized implementations obviously result in reduced accuracy, which is more noticeable in the case of the FPGA implementation with the MATLAB^®^ Deep Learning HDL Toolbox^TM^ but is almost negligible for Google Coral.

### 5.2. Performance

**Figure 10:**
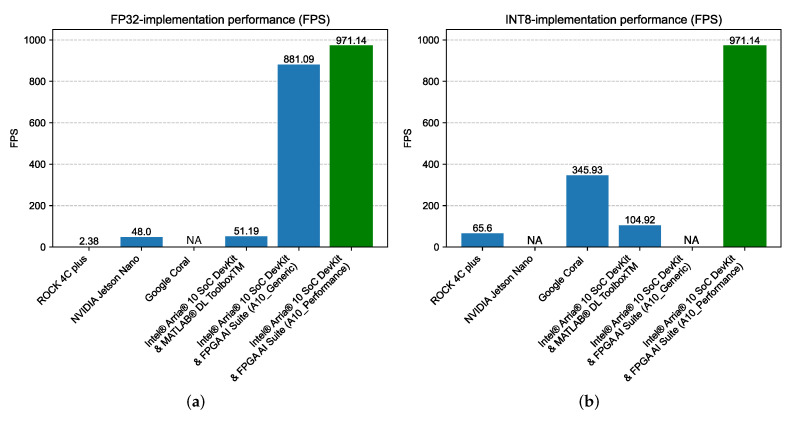
Graphical performance comparison for (**a**) FP32 implementations and (**b**) INT8-quantized implementations (performance of the A10_Performance implementation for the Intel^®^ Arria^®^ 10 SoC DevKit and Intel^®^ FPGA AI Suite is shown in green as a benchmark).

The same AI application was executed on all the devices, with the same quantization and operation conditions, to ensure a reliable performance comparison among the different edge devices. The time required for predicting all the samples was averaged to calculate latency and frames-per-second (FPS) as performance metrics. The performance results of all devices and implementations under test are summarized in Table 6, and are graphically compared in Figure 10. Thus, several interesting conclusions can be drawn. First, FPGA-based solutions provide the best performance, using the Intel^®^ FPGA AI Suite implementations. Their overall throughput is clearly superior to any of the other alternatives, with an approximately ×20 increase in performance when the Intel^®^ FPGA AI Suite A10_Performance architecture is compared with the MATLAB^®^ Deep Learning HDL Toolbox^TM^ FP32 implementation or the NVIDIA Jetson Nano. Second, while the TPU-accelerated Google Coral option provides a remarkable performance when INT8 quantization is considered, its performance is still around a third of what is possible with the Intel^®^ Arria^®^ 10 SX SoC Development Kit. This remarkable performance of the FPGA devices enables real-time computing of the RIS configuration, making it suitable for a broader range of applications that require more demanding computing capabilities. At the same time, it makes it possible to embed the RIS configuration problem into an edge computing system, since the FPGA may host a more complex system along with the AI accelerator, which in this particular RIS application may include the control system, additional accelerators for RIS management or application, or even hardware not directly related to the RIS. On an additional note, while the use of the MATLAB^®^ Deep Learning HDL Toolbox^TM^ does not provide the best performance, it is interesting to note that the development of this kind of implementation does require practically no hardware background from the user and makes it possible to integrate the AI accelerator within the MATLAB^®^ ecosystem for further development of the global application. Finally, it must be noted that the performance advantages shown here for the FPGA-based implementations were obtained using HLS tools. This opens the possibility for the development of custom accelerators that could further improve the performance of AI hardware, especially when new AI-oriented device families are used as target devices.

**Table 5:** Accuracy comparison of NN execution across the different devices and implementations.

Devices	FP32	INT8
**ROCK 4C plus**	98.88%	98.56%
**NVIDIA Jetson Nano**	98.88%	-
**Google Coral**	-	98.44%
**Intel^®^ Arria^®^ 10 SoC DevKit & MATLAB^®^ DL Toolbox^TM^**	98.88%	91.62%
**Intel^®^ Arria^®^ 10 SoC DevKit & FPGA AI Suite (A10_Generic)**	98.88%	-
**Intel^®^ Arria^®^ 10 SoC DevKit & FPGA AI Suite (A10_Performance)**	98.88%	-

**Table 6:** Performance comparison of NN execution across the different devices and implementations.

	FP32		INT8	
Device	FPS	Latency (ms)	FPS	Latency (ms)
**ROCK 4C plus**	2.38	420.17	65.60	15.24
**NVIDIA Jetson Nano**	48.00	20.83	-	-
**Google Coral**	-	-	345.93	2.89
**Intel^®^ Arria^®^ 10 SoC DevKit & MATLAB^®^ DL Toolbox^TM^**	51.19	19.54	104.92	9.53
**Intel^®^ Arria^®^ 10 SoC DevKit & FPGA AI Suite (A10_Generic)**	881.09	1.13	-	-
**Intel^®^ Arria^®^ 10 SoC DevKit & FPGA AI Suite (A10_Performance)**	971.14	1.03	-	-

### 5.3. Analysis of Resource Usage and Performance for FPGA Implementations

Since the FPGA device has been shown to provide the best performance, as detailed above, it is interesting to analyze the resource usage for the different implementations in the Intel^®^ Arria^®^ 10 SoC DevKit. Thus, Table 7 summarizes the resource usage for the two implementations carried out for each of both MATLAB^®^ Deep Learning HDL Toolbox^TM^ and Intel^®^ FPGA AI Suite options. In this table, the resource usage of the A10_Peformance architecture with the Intel^®^ FPGA AI Suite is used as the benchmark data, as it provides the best performance in Table 6. Therefore, the resource usage for the other implementations is also shown as percentages (BM %) of the resources for this benchmark. It is clear from these data that the Intel^®^ FPGA AI Suite provides more efficient implementations than the MATLAB^®^ Deep Learning HDL Toolbox^TM^, not only in terms of performance but also in terms of resource usage, especially when comparing the number of required embedded memory blocks (BlockRAM) and logic elements (ALM). It is also worth noting the difference in device occupation between the two MATLAB^®^ Deep Learning HDL Toolbox^TM^ implementations: while the INT8-quantized version implies a slight reduction in accuracy, it is able to almost double the performance over the FP32 option thanks to a more intensive use of ALMs and, particularly, the embedded-multiplier variable-precision DSP blocks. In any case, resource utilization for any of the Intel^®^ FPGA AI Suite options leaves more than half of the FPGA device available for implementing additional logic. In this way, the FPGA implementation of the AI-based configuration of an RIS becomes a viable edge computing system, as the FPGA can still host any other required logic for RIS control, communications, or other related tasks, while maintaining the performance of the CNN computation.

## 6. Conclusions

This work shows a novel approach to obtain the optimal configuration of a binary RIS using AI techniques. The input data to train and compute the desired configuration are an image representing the target redirection angle, and, therefore, a custom CNN is proposed to implement the problem solution. The resulting model underwent evaluation on various computing devices, including a CPU, a TPU, a GPU, and an FPGA, with the ultimate aim of performing optimal RIS configuration computations at the edge. The evaluation was conducted under identical conditions for each device, implementing the NN with the FP32 and INT8 data types in order to facilitate performance comparisons. The FPGA was initially considered the most suitable device for NN inference, due to its inherent reconfigurability and adaptability to different algorithms and use cases. As expected, all devices produced comparable accuracy results under the same conditions, but there were significant variations in terms of performance. While TPUs and GPUs offer remarkable performance, the FPGA-based implementations exhibited a ×20 performance increase over the closest FP32 option, with a performance of 971 FPS compared with the 48 FPS achieved with the NVIDIA Jetson Nano. If the INT8-quantized, TPU-accelerated implementation on the Google Coral is considered in the comparison, despite its inferior accuracy, the FPGA-based implementations still offer an almost ×3 performance advantage (971 FPS vs. 346 FPS). Furthermore, for the implementation of the neural network on FPGA devices, the use of innovative HLS tools significantly reduces development time and simplifies the overall design process, making it especially suitable for rapid prototyping while achieving high performance. However, it is worth noting that further performance enhancements can be achieved on FPGA devices once new AI-oriented device families and custom DL accelerators, beyond what is possible with HLS tools, are introduced.

## Figures and Tables

**Table 1:** Summary of network model.

Layer	Type	Output Shape	Activation	Number of Parameters
Input	Padding	[360,360,3]	-	0
Lambda ^1^	Lambda ^1^	[360,360,3]	-	0
Conv2d_0	Conv2D	[180,180,8]	ReLU	224
Conv2d_1	Conv2D	[90,90,16]	-	64
BN_0	BatchNormalization	[90,90,16]	-	64
LR_0	Activation Layer	[90,90,16]	ReLU	0
Conv2d_2	Conv2D	[45,45,32]	ReLU	4640
Conv2d_3	Conv2D	[15,15,64]	-	18,496
BN_1	BatchNormalization	[15,15,64]	-	64
LR_1	Activation Layer	[15,15,64]	ReLU	0
Conv2d_4	Conv2D	[8,8,128]	ReLU	73,856
Conv2d_5	Conv2D	[4,4,256]	ReLU	131,328
Conv2d_6	Conv2D	[1,1,512]	ReLU	2,097,664
FC_0	Dense	[1,1,256]	ReLU	131,328
FC_1	Dense	[1,1,225]	Sigmoid	57,825
Reshape	Reshape	[15,15,1]	-	0
**Total:**				**2,516,849 **

^1^ The Lambda layer applies a normalization to the original image.

**Table 2:** ROCK 4C Plus, NVIDIA Jetson Nano, Google Coral, and Intel^®^ Arria^®^ 10 SX SoC Development Kit specification summary.

	ROCK 4C Plus	NVIDIA Jetson Nano	Google Coral	Intel^®^ Arria^®^ 10 SX SoC Development Kit
**Device Family**	CPU + GPU	GPU + CPU	Edge TPU	FPGA + CPU
**CPU **	ARM^®^ Cortex^™^-72 + ARM^®^ Cortex^™^-A53	ARM^®^ Cortex^™^-A57	ARM^®^ Cortex^™^-A53 + ARM^®^ Cortex^™^-M4	ARM^®^ Cortex^™^-A9 MPCore
**CPU Cores **	2 + 4	4	4 + 1	2
**CPU Architecture **	64-bit	64-bit	64-bit	32-bit
**CPU Max. Freq. **	1.5/1.0 GHz	1.43 GHz	1.5 GHz	1.2 GHz
**AI Acceleration **	-	NVIDIA Maxwell architecture with 128 NVIDIA CUDA^®^ cores	Google Edge TPU coprocessor	FPGA with 251,680 ALMs + 1687 Variable Precision DSPs + 2131 20-kb BlockRAMs
**RAM Memory **	4 GB 64-bit LPDDR4 3200 MHz	4 GB 64-bit LPDDR4 1600 MHz	4 GB 32-bit LPDDR4 1600 MHz	2 GB + 1 GB 16-bit DDR4 1200 MHz
**Framework **	TensorFlow	TensorFlow	TensorFlow Lite	MATLAB^®^ Deep Learning HDL Toolbox^TM^/Intel^®^ FPGA AI Suite
**Operating System **	Debian Desktop 5.10.110	Ubuntu Desktop 4.9.253	Mendel Linux 4.14.98	Linux Intel SoC 4.9.0/Yocto Linux 5.15.70

**Table 3:** Resource usage in Intel^®^ Arria^®^ 10 SX SoC Development Kit with MATLAB^®^ Deep Learning HDL Toolbox^TM^.

		ALM	BlockRAM Memory Bits	BlockRAM	Variable Precision DSP
	**Total**	251,680	43,642,880	2131	1687
**FP32**	**Used**	134,187	23,133,724	2131	255
	**Usage %**	53.32%	53.01%	100.00%	15.12%
**INT8**	**Used**	160,818	17,584,432	2131	730
	**Usage %**	63.90%	40.29%	100.00%	43.27%

**Table 4:** Resource usage in Intel^®^ Arria^®^ 10 SX SoC Development Kit with Intel^®^ FPGA AI Suite.

		ALM	BlockRAM Memory Bits	BlockRAM	Variable Precision DSP
	**Total**	251,680	43,642,880	2131	1687
**A10_Generic**	**Used**	48,899	14,371,616	777	182
	**Usage %**	19.42%	32.93%	36.46%	10.79%
**A10_Performance**	**Used**	68,624	20,452,640	1102	606
	**Usage %**	27.26%	46,36%	51.71%	35.92%

**Table 7:** Resource usage comparison in Intel^®^ Arria^®^ 10 SX SoC Development Kit.

		ALM	BlockRAM Memory Bits	BlockRAM	Variable Precision DSP
**Intel^®^ FPGA AI Suite A10_Performance**	**Used**	68.624	20,452,640	1102	606
**MATLAB^®^ DL** **Toolbox^TM^ FP32**	**Used** **BM %**	134,187 195.54%	23,133,724 113.11%	2131 193.38%	255 42.08%
**MATLAB^®^ DL** **Toolbox^TM^ INT8**	**Used** **BM %**	160,818 234.35%	17,584,432 85.98%	2131 193.38%	730 120.46%
**Intel^®^ FPGA** **AI Suite** **A10_Generic**	**Used** **BM %**	48,899 71.26%	14,371,616 70.27%	777 64.15%	182 30.03%

## Data Availability

Data are contained within the article.
